# A Hybrid RSM-ANN-GA Approach on Optimization of Ultrasound-Assisted Extraction Conditions for Bioactive Component-Rich *Stevia rebaudiana* (Bertoni) Leaves Extract

**DOI:** 10.3390/foods11060883

**Published:** 2022-03-20

**Authors:** Kashif Ameer, Saqib Ameer, Young-Min Kim, Muhammad Nadeem, Mi-Kyung Park, Mian Anjum Murtaza, Muhammad Asif Khan, Muhammad Adnan Nasir, Ghulam Mueen-ud-Din, Shahid Mahmood, Tusneem Kausar, Muhammad Abubakar

**Affiliations:** 1 Institute of Food Science and Nutrition, University of Sargodha, Sargodha 40100, Pakistan; nadeem.abdul@uos.edu.pk (M.N.); anjum.murtaza@uos.edu.pk (M.A.M.); ghulam.mueen@uos.edu.pk (G.M.-u.-D.); shahid.mustafa@uos.edu.pk (S.M.); tusneem.kausar@uos.edu.pk (T.K.); 2 Department of Computer Science & Information Technology, Faculty of Information Technology, The University of Lahore, Lahore Campus, Defense Road, Lahore 54000, Pakistan; saqib1430@gmail.com; 3 Department of Food Science & Technology, Chonnam National University, Gwangju 61186, Korea; 4 School of Food Science and Biotechnology, Kyungpook National University, Daegu 41566, Korea; parkmik@knu.ac.kr; 5 Department of Food Science and Technology, Islamia University of Bahawalpur, Bahawalpur 63100, Pakistan; asifkhan.muhammad@gmail.com; 6 University Institute of Diet and Nutritional Sciences, The University of Lahore, Gujrat Campus, Gujrat 50700, Pakistan; adnan.nasir@dnsc.uol.edu.pk; 7 Department of Allied Health Sciences, The University of Chenab, G.T. Road, Gujrat 50700, Pakistan; 8 Department of Electrical Engineering, Quaid-e-Azam College of Engineering & Technology, Sahiwal 57000, Pakistan; mabubakarqazi@gmail.com

**Keywords:** *Stevia rebaudiana*, ultrasound-assisted extraction, stevioside, rebaudioside-A, optimization

## Abstract

*Stevia rebaudiana* (Bertoni) leaves consist of dietetically important diterpene steviol glycosides (SGs): stevioside (ST) and rebaudioside-A (Reb-A). ST and Reb-A are key sweetening compounds exhibiting a sweetening potential of 100 to 300 times more intense than that of table sucrose. Ultrasound-assisted extraction (UAE) of SGs was optimized by effective process optimization techniques, such as response surface methodology (RSM) and artificial neural network (ANN) modeling coupled with genetic algorithm (GA) as a function of ethanol concentration (*X*_1_: 0–100%), sonication time (*X*_2_: 10–54 min), and leaf–solvent ratio (*X*_3_: 0.148–0.313 g·mL^−1^). The maximum target responses were obtained at optimum UAE conditions of 75% (*X*_1_), 43 min (*X*_2_), and 0.28 g·mL^−1^ (*X*_3_). ANN-GA as a potential alternative indicated superiority to RSM. UAE as a green technology proved superior to conventional maceration extraction (CME) with reduced resource consumption. Moreover, UAE resulted in a higher total extract yield (TEY) and SGs including Reb-A and ST yields as compared to those that were obtained by CME with a marked reduction in resource consumption and CO_2_ emission. The findings of the present study evidenced the significance of UAE as an ecofriendly extraction method for extracting SGs, and UAE scale-up could be employed for effectiveness on an industrial scale. These findings evidenced that the UAE is a high-efficiency extraction method with an improved statistical approach.

## 1. Introduction

*Stevia rebaudiana* (Bertoni) is classified as a shrub belonging to the Asteraceae family that originated primitively from the Amambay region of Paraguay. Stevia as a non-caloric natural sweetener has a long history of use in various parts of the world as a substitute for artificial sweeteners and sucrose such as, Brazil, China, South Korea, and Japan [1,2]. In these days, the concerns pertaining to adverse health effects of artificial sweeteners, such as Acesulfame-K and Saccharin have been on verge of rise and have fostered the attempts to explore alternative natural sweetening compounds [3,4]. 

*S. rebaudiana* (Bert.) contains a wide variety of several important phytochemicals: diterpenes, triterpenes, labdane, stigmasterol, volatile oils, tannins, and a total of eight steviol glycosides (SGs). These diterpene glycosides are dulcoside, stevioside (ST), steviolbioside, rebaudioside-A (Reb-A), Reb-B, Reb-C, Reb-D, and Reb-E. ST and Reb-A have 200–300 and 400 times higher sweetness intensities, respectively, when compared with normal table sugar (sucrose) [5]. Back in 2011, the European Food Safety Authority (EFSA) gave approval to stevia as a food additive and non-caloric sweetener with an approved acceptable daily intake (ADI) of 4 mg/kg BW for steviol glycosides (SGs). Similarly in 2008, the U.S. Food and Drug Administration (FDA) gave approval to stevia glycosides for usage in the form of a highly purified extract by granting it a Generally Recognized as Safe (GRAS) status [2,6]. 

The classical extraction methods of SGs have also been reported in various published reports; stir-aided classic thermal extraction, decoction and infusion [7], conventional cold solvent extraction via different extractants [8], sorbitol, glycerin, and propylene glycol [9], hot water extraction [10], maceration extraction (cold and hot), Soxhlet extraction, hydro-distillation [11], water extraction [12], and traditional mix-stirring [13]. However, conventional extraction approaches are laborious, less efficient, and possess certain disadvantages namely lower extract yields of target compounds, high usage of solvents, time and energy, thermo-induced deterioration of the bioactive components in plant matrices, in conjunction with volatile compounds loss at elevated temperatures [14]. By contrast, modern green extraction techniques, i.e., supercritical fluid extraction, ultrasound-assisted extraction (UAE), and microwave-assisted extraction offer improved selectivity, increased stability, and render clean extracts with adequate organoleptic properties [14]. Among these, UAE has emerged as a promising technique that is efficient, simple, and economically cost-effective. These techniques positively impact the efficiency by increasing one or more prominent extraction parameters governing the extraction mechanisms: interfacial region, extractions kinetics, and the mass transfer of solutes from the plant matrices [14,15].

Response surface methodology (RSM) as a process optimization technique involves the exploitation of sophisticated mathematical and statistical techniques for the development, improvement, and optimization of the processes. Artificial neural network (ANN), which is inspired from the functioning of the biological brain, has emerged as a powerful non-linear computational technique for complex non-linear processes owing to its strong learning and predictive modeling capacities [1,16]. The genetic algorithm is one of the optimization algorithms that is known as the heuristic approach inspired from the concept of “survival of the fittest” and is widely reported to yield feasible optimal solutions with a known fitness function. The depiction of the fitness value vs generation plot for the genetic algorithm are shown in Figure S1, whereas the setting parameters of genetic algorithm that are used in the optimization of process are provided in Table S1. The hybrid ANN-GA method is usually implemented by employing the RSM-ANN data points for algorithm initialization. In a recent report by Yahya et al. [17], it was implied by researchers that the application of ANN-GA led to achieve higher predictive accuracy with more proximity to the experimental data in comparison with those of RSM-predicted values. Pertaining to the best of our information and knowledge in the open literature, there is no research report that is available currently with respect to ANN and RSM comparison for optimizing UAE of ST and Reb-A from *S. rebaudiana* (Bert.) leaves. Previous research was carried out to optimize the UAE condition for steviol glycosides extraction, however no structured statistical approach was employed [18] and no numerical data trend was analyzed by further applying multivariate techniques, such as principal component analysis (PCA) and RSM. Moreover, in another report, only RSM was utilized to optimize the UAE extraction conditions for steviol glycosides but the authors did not compare the predictive modeling efficiency with improved approaches, such as ANN and GA [19]. This is probably the first report about using a hybrid RSM-ANN-GA approach on the optimization of UAE conditions for bioactive components-rich *Stevia rebaudiana* (Bertoni) leaves extract. The need to intensify the extraction of th main bioactive sweetening compounds such as Reb-A and ST from *S. rebaudiana* (Bert.) leaves has led to explore the optimization of the UAE process. UAE extraction conditions require improvement, and modeling of UAE is critical for identifying the optimum extraction conditions that are suited to market factors. Water and ethanol as extraction solvents have been reported in published literature [20]. However, ethanol as an extraction solvent has been preferred by some researchers for SGs extraction owing to its GRAS status; recovery of higher extraction yields because of the presence of a hydroxyl group in organic solvents of polar nature, such as ethanol; and it has been also exploited as a green solvent for bioactive components extraction from plant matrices and high quality foods, such as pigments, resins, antioxidants, resins, and essential oils etc. [21].

This study was aimed at the employment of the CCD design configuration (five-level-three-factor) of RSM and an ANN-based model development along with comparison to optimize the UAE process parameters to elucidate the influence of independent process variables on target sweetening compounds recovery—total extract yield (TEY), ST, and Reb-A yields from matrix o stevia leaf powder and this can be achieved at optimum UAE conditions. The CCD based-independent practical variables such as the concentration of ethanol (*X*_1_), UAE-assisted sonication time (*X*_2_), and the ratio of leaf to solvent (*X*_3_) were employed for the determination of process parameter effects on the recovery of extract and sweetening compounds (ST and Reb-A). Experimental data that were obtained from the CCD configuration at specified design points was utilized to develop the ANN-GA model to get the best possible optimized solutions. Moreover, the conventional maceration and UAE extraction methods were compared in terms of the obtained TE, ST, and Reb-A yields; energy consumption; and CO_2_ emission. Therefore, a hybrid RSM-ANN-GA approach was employed for the optimization of UAE conditions for bioactive component-rich *Stevia rebaudiana* (Bertoni) leaves extract.

## 2. Materials and Methods

### 2.1. Stevia Leaf Powder Preparation, Reagents and Chemicals

The dried stevia leaves of Vietnamese origin (harvested in 2015) were procured from the Daepyung Co., Pvt. Ltd. (Hamchang-Eup, Sangju-Si, Gyeongsangbuk-Do, Korea). A fine leaf powder was obtained by grinding through use of dry grinder (Lab-scale, FM-909 T, Hanil Electric Co., Seoul, Korea). Polythene bags were used for tightly packing the finely ground leaf powder followed by storage at −10 °C until further experiments.

### 2.2. Conventional Maceration Extraction Procedure

As the control method, CME was carried out as per the methodology details in the reported method of Alupului et al. [7]. The stevia leaf powder was taken in a quantified amount of 10 g followed by mixing in a closed Erlenmeyer flask with distilled water (300 mL). Then, the mixture was subjected to standing time of 24 h at ambient room temperature (28 °C). After the completion of standing time, the crude extract was filtered with Whatman Filter Paper No. 41 (GE Healthcare, Buckinghamshire, UK) and then Falcon tubes (50 mL) were used to keep the clear extracts. Then, the tubes were subjected to storage at after tightly closing the tubes caps at 4 ± 1 °C storage temperature until further analyses.

### 2.3. Ultrasound Assisted Extraction Procedure

UAE of *S. rebaudiana* (Bert.) was completed procedurally in accordance with the method of Liu et al. [13] with some modifications by using a microprocessor-controlled bench-top sonication cleaning bath (Powersonic-420, Hwashin Tech. Co. Seoul, Korea). Subsequently, the extraction was carried out by placing the flask in the ultrasonic bath at ambient room temperature (28–30 °C) in accordance CCD-specified conditions. After UAE, the liquid extracts were subjected to vacuum filtration under reduced pressure and then filtration was performed using Whatman filter paper No. 41 followed by pouring the clean extracts in Falcon tubes (50 mL). Then, the tubes were subjected to storage after tightly closing the tubes caps at 4 ± 1 °C storage temperature until further analyses.

### 2.4. Preliminary Screening Study and RSM-Based Experimental Design

**Figure 1:**
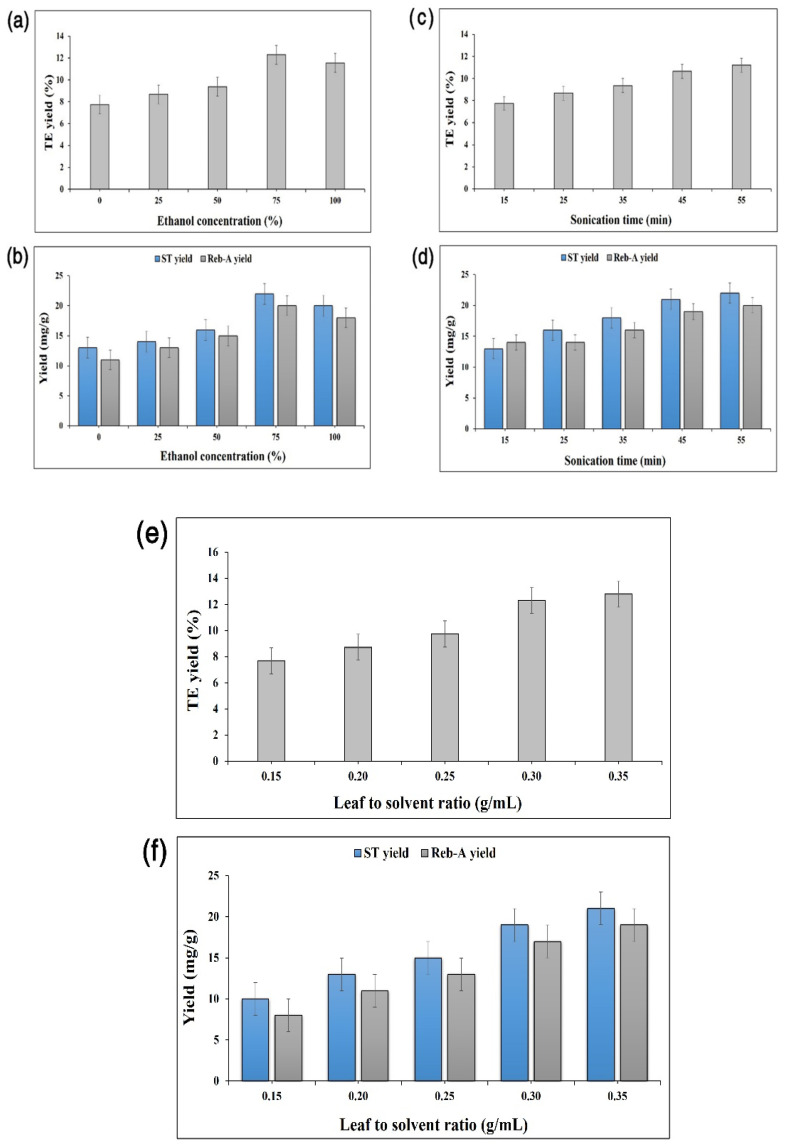
Effect of *X*_1_ (ethanol concentration) on TEY (%) target response, (**a**) the effect of *X*_1_ (ethanol concentration) on ST and Reb-A yields (mg/g), (**b**) ST yield: sonication time effect on TEY (%) target response, (**c**) sonication time effect on ST and Reb-A yields (mg/g), (**d**) Leaf-to-solvent ratio effect on the TEY (%) response, (**e**) Leaf-to-solvent ratio effect on ST and Reb-A yields (mg/g) responses (**f**).

A preliminary screening study was carried out for determining the appropriate ranges of independent UAE process variables: ethanol concentration (*X*_1_), sonication time (*X*_2_), and leaf–solvent ratio (*X*_3_) on the basis of a literature review [13,22,23]. *X*_1_ was varied from 0 to 100% by keeping *X*_2_ and *X*_3_ fixed at 32 min and 0.23 g·mL^−1^, respectively. Similarly, *X*_2_ was changed within a range of 10–54 min, while both *X*_1_ and *X*_3_ were kept fixed at 50% and 0.23 g·mL^−1^, respectively. Similarly, X_3_ was varied from 0.148 to 0.313 g·mL^−1^, while *X*_1_ and *X*_2_ were subjected to fixing at defined levels of 50% and 32 min, respectively. The preliminary experimental results that are shown in Figure 1 revealed increases in the response variables (*Y*_1_: TEY, *Y*_2_: ST yield, and *Y*_3_: Reb-A yield) with corresponding rises in the input variables, and hence, were selected as the most influential parameters. All the UAE experimental runs were carried out in accordance with the specifications that were laid down by a 3–factor–5–level CCD. Moreover, all the independent process parameters were varied over 5 levels (−α, −1, 0, +1, +α) with the variables coding according to Equation (1):

(1) xi=Xi−XcpΔXi  where i=1,2,3,….., k 

In Table 1, the description of the input UAE process variables with their particular experimental ranges is given, along with their units and coded notations (Equation (2))

(2) $$Y=\beta_{0}+\overset{n}{\underset{i=1}{\sum }}\beta_{i}X_{i}+\overset{n-1}{\underset{\begin{array}{c} i=1\\ j>1 \end{array}}{\sum }}\overset{n}{\underset{j=2}{\sum }}\beta_{ij}X_{i}X_{j}+\overset{n}{\underset{i=1}{\sum }}\beta_{ii}X_{i}^{2}+\varepsilon \;$$

where *Y* designates the target responses, whereas *β*_0_ denotes the constant coefficient. *β_i_*, *β_ii_*, and *β_ij_* are indicative of the regression coefficients pertaining to the UAE process variables corresponding to linear, quadratic, and interaction effects, respectively. The independent UAE process variables are presented by *X_i_* and *X_j_*, respectively, and ε depicts the error.

### 2.5. Artificial Neural Network (ANN) Modeling

**Figure 2:**
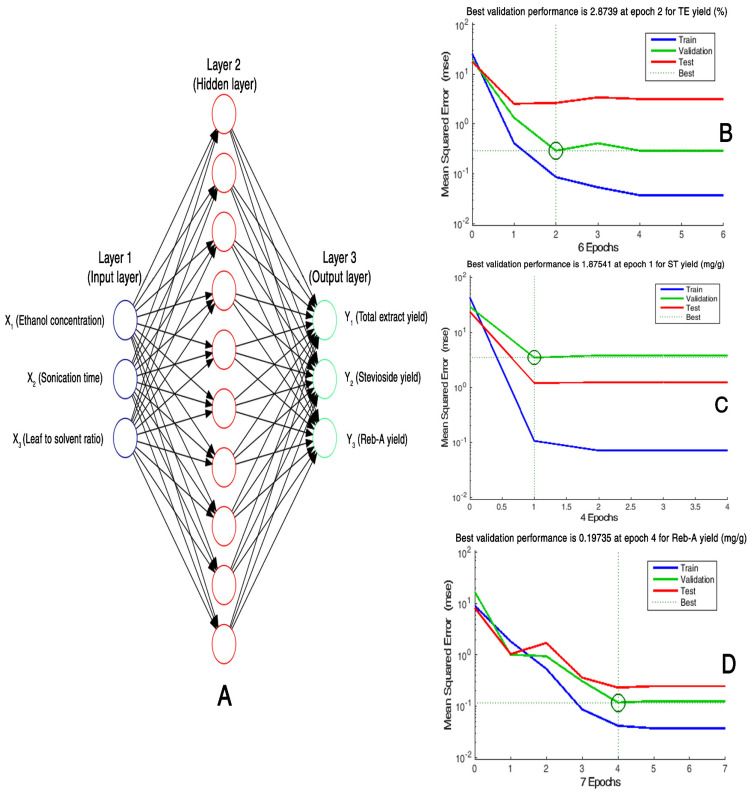
Optimal architecture of the multiplayer perceptron (MLP) topology of the developed ANN model. (**A**) Depiction of the network training curves demonstrating the number of Epochs for the trained subsets for TEY (%) target response, (**B**) ST yield (mg/g), (**C**) and Reb-A yield (mg/g) target responses (**D**).

ANN is a powerful modeling tool owing to its learning and generalization capabilities for approximating complex behaviors of non-linear processes. Multilayer perceptron (MLP), a feed-forward ANN architecture with back propagation (BP) algorithm, was chosen because of its capacity to model any function [1], and subsequently trained in order to map the input layer (*X*_1_, *X*_2_, *X*_3_) and output layer (*Y*_1_, *Y*_2_, *Y*_3_) for the development of a predictive model. Figure 2A shows a three-layered topology (architecture) of an ANN model. The manipulation of the ANN architecture was carried out by varying the neuron number in the hidden layer. It comprised of 3 layers: input, hidden, and output layers. The ANN model topology was specified in terms of 3-h-3; whereby 3 neurons of the input layer corresponded to 3 input variables, h was designated to the number of neurons in a single hidden layer, and 3 neurons in the output layer corresponded to the target responses. The number of neurons in the input and output layers was defined by the corresponding number of input (*X*_1_, *X*_2_, *X*_3_) and output (*Y*_1_, *Y*_2_, *Y*_3_) variables, respectively. The same set of experimental data that were employed for RSM modeling were subjected to the simulation by ANN modeling. Using the experimental data, building to various network topologies was performed followed by training, testing, and subsequently validation through variation in the number of hidden layers over a range from 1 to 10, and a number of neurons in the hidden layer from 1 to 15 was also varied with the sole objective to minimize the degree of deviations between the experimental and predicted values. A feed-forward ANN comprising MLP with BP algorithm was developed by using the Neural Network Toolbox™ of MATLAB R2015b. An entire experimental dataset was divided into 3 sets that were named training, validation, and training. Data points proportion that were employed for various purposes was designated as follows: 70% (14 points) for training of network, 15% (3 points) to validate the developed model architecture, and the leftover 15% (3 points) was employed to fulfill the testing purpose. In this study, the sigmoid transfer function was employed at the hidden layer and a linear transfer function was used for activating the neurons at the process parameters (input) and target responses (output) layers. A trial and error searching method was used to carry out the training process until the attainment of minimum mean square error (MSE) during the validation process.

### 2.6. Genetic Algorithm

The data that were obtained from the developed ANN network was employed as the initial population using a genetic algorithm (GA) through RStudio software (RStudio Community, Boston, MA, USA). “R” and its libraries implement a wide variety of statistical and graphical techniques, including linear and nonlinear modeling, classical statistical tests, time-series analysis, classification, clustering, and others [24]. The following R packages were utilized to complete the GA optimization; Tidyverse, GA, Ranger, Tidymodels, Caret, and Tictoc packages. As an iterative and population-based global search optimization algorithm, GA has been widely utilized as a hybrid approach with ANN to optimize non-linear complex problems. While implementing a hybrid ANN-GA algorithm for optimization, several steps are involved, such as initialization; selection pertaining to fitness evaluation followed by genetic operators, such as reproduction, crossover and mutation; and all these steps are sequentially performed until the obtainment of optimal solutions [25]. Regarding the setup related to the problem under evaluation, GA was subjected to selection using different functions, such as the solver and fitness functions from ANN, that were employed as the objective function in GA implementation for achieving maximum values of all the target responses. The further optimization endorsement of the UAE conditions was carried out through the use of a genetic algorithm (GA) by employing the RSM-ANN-generated dataset as the initial population. In the case of employing the objective function in GA implementation, it is imperative to utilize only the scalar values instead of the first- or second-order functional derivatives. The network data from the developed and trained RSM-ANN was trained for the objective function, and GA was implemented with maximization of the problems. 

### 2.7. Determination of Total Extract Yield (TEY)

TEY was calculated by using the reported method of Ameer et al. [1] with some modifications. A tarred round bottom flask was utilized for transferring the obtained extract followed by evaporation by means of a rotary evaporator that was operated under the vacuum condition. Then, a hot air oven was used to dry the flask at 105 °C until complete dryness to a constant weight followed by weights calculation using the following Equation (3):

(3) $$Total\;extract\;yield\;\left(\%\right)=\frac{A\;–\;B}{W}\times 100\;$$

where, *A* represents the constant weight of flask with sample after oven drying, *B* denotes the empty dry flask weight, whereas *W* denotes the total sample weight.

### 2.8. HPLC Analysis

The sample preparation for HPLC analysis was procedurally completed as per the details that were described by Erkucuk, Akgun, and Yesil-Celiktas [26]. HPLC quantification of SGs including ST and Reb-A was carried out in accordance with the international standard guidelines that were specified by Joint FAO/WHO Expert Committee on Food Additives (JECFA) approved at the 69th meeting being held in Geneva and were published in the FAO/JECFA monograph [27]. Agilent-1260 HPLC system (Agilent Tech., Santa Clara, CA, USA) with UV detector (210 nm) was employed for the detection and quantification of target steviol glycosides (ST and Reb-A) in the UAE extracts. The column named TSKgel Amide–80 column (4.6 mm ID, 250 mm length, and 5 μm particle size) that was supplied by Tosoh Bioscience Corp., Tokyo, Japan was employed to separate the ST and Reb-A. Maintenance of column temperature and was kept at the ambient room temperature of 25 °C in order to carry out the HPLC analysis. A mobile phase consisting of a mixture of acetonitrile and water (80:20 *v*/*v*) was used for chromatographic separation of SGs at a flow rate of 1 mL min**^−^**^1^. The mobile phase pH was maintained at a specific value of 3 using phosphoric acid (5.9 N). A sample volume of 20 μL was injected during all the runs. SGs including ST and Reb-A percentages were calculated through the JECFA-specified formula for all steviol glycosides as shown below in Equations (4) and (5).

(4) $$X\;\left(\%\right)=\left[\frac{W_{s}}{W}\right]\times \left[\frac{f_{x}A_{x}}{A_{s}}\right]\times 100\;$$

In this equation, *X* expresses the percentage of ST, *Ws* represents the ST dry weight in milligrams present in standard solution, and *W* represents the sample’s dry weight (mg) in the sample solution. *A_x_* and *A_s_* represent the peak areas of ST from the sample and standard solutions, respectively. *f_x_* corresponds to the ratio of molecular weight of steviol glycoside (*X*) to molar mass of ST (804.872 g/mol) or Reb-A (967.013 g/mol).

Reb-A that was present in sample solution was quantified by using the following formula in accordance with the protocol that was published in the FAO/JECFA monograph.

(5) $$Reb-A\;\left(\%\right)=\left[\frac{W_{R}}{W}\right]\times \left[\frac{A_{x}}{A_{R}}\right]\times 100\;$$

In this equation, *W_R_* represents the dry weight of Reb-A (mg) that was present in the standard solution while *W* is the dry weight of the sample (mg) in the sample solution. *As* in the case of *ST*, and *A_R_* and *A_x_* represent the Reb-A peak areas from the standard and sample solutions, respectively.

### 2.9. Statistical Analysis

The Optimization Toolbox™ (for implementing second-order polynomial central composite design of RSM), Neural Network Toolbox™ (Feed-forward ANN comprising MLP with BP algorithm implementation) of MATLAB R2015b software (The Mathworks, Inc., Ver. 8.6.0.347, MA, USA), and Microsoft Excel 2013 (15.0.44) (Microsoft Corporation, Redmond, WA, USA) were used for carrying out the one-way analysis of variance (ANOVA) and differences between the means were calculated using a Duncan multiple range test at significance level of *p* < 0.05.

#### Performance Comparison of RSM and ANN-GA Models

The predictive performance assessment of the employed modeling approaches including *RSM* and ANN-GA models were subjected to analysis through the use of different statistical indicators; coefficient of determination (*R*^2^), root mean square error (*RMSE*), the absolute average deviation (*AAD*), and the standard error of prediction (*SEP*) (Equations (6)–(9)) [28,29,30].

(6) $$RMSE={\left(\frac{1}{n}\;\overset{n}{\underset{i=1}{\sum }}{\left(Y_{predict}\;–\;Y_{exp}\right)}^{2}\right)}^{\frac{1}{2}}\;$$

(7) $$R^{2}=\frac{{\left(\sum_{i=1}^{n}\left(Y_{exp}-\bar{Y_{exp}}\right)\;\left(Y_{predict}-\bar{Y_{predict}}\right)\right)}^{2}}{\sum_{i=1}^{n}{\left(Y_{exp}-\bar{Y_{exp}}\right)}^{2}\;{\left(Y_{predict}-\bar{Y_{predict}}\right)}^{2}}\;$$

(8) $$AAD\;\left(\%\right)=\left[\frac{\sum_{i=1}^{p}\left(\left|Y_{i,exp}-Y_{i,cal}\right|/Y_{i,exp}\right)\;}{P}\right]\times 100$$

(9) SEP (%)=RMSEYe ×100 

whereby the number of sample points are denoted by *n*, the predicted response value is designated by *Y_predict_*, whereas *Y_exp_* is indicative of the experimental value, and “−” over variables represents the average value of the concerned variable values. 

## 3. Results and Discussion

### 3.1. RSM Modeling of UAE Process

All of the UAE experiments were performed in terms of triplicate manner in accordance with the CCD matrix specifications as shown in Table 2 and data analysis was performed by considering the mean experimental values to obtain good model fitting and second-order quadratic model equations (Equations (10)–(12)). Statistical significance and adequacy of these model equations were evaluated by using analysis of variance (ANOVA) (Table 3). For the fitted model, further evidence pertaining to the goodness of fit was provided by the model summary statistics and the model demonstrated high significance as evident from the lower probability values (*p* < 0.0001), high *R*^2^, adjusted *R*^2^ values, along with the predicted *R*^2^ values. The *R*^2^ and *p*-values of Equations (10)–(12) were 0.9401; 0.0812, 0.8874, and 0.1356; and 0.9758; and 0.09517, respectively. The ANOVA results demonstrated that linear, quadratic, and interactive coefficients were significant owing to the lower *p*-values and higher F-values and had considerably large effects on TE and SGs (ST and Reb-A) yields from the UAE extracts. Moreover, the validity of the quadratic model was endorsed in terms of a non-significant lack of fit (>0.05) values with better precision and reliability of the developed model. Three-dimensional (3D) surface plots were constructed based on polynomial regression equations in order to elucidate the interaction effects of the input variables of the UAE process on the response variables (*Y*_1_, *Y*_2_, *Y*_3_).

### 3.2. Process Variables Effect on Total Extract Yield (YEY)

TEY values of the UAE*–*derived extracts were presented in Table 2 with the corresponding extraction conditions according to the CCD matrix. The coded form using coefficients is shown in Table 3 as shown in Equation (10):

(10) $$Y_{1}\;\left(\%\right)=+26.8068-0.3350X_{1}+0.4716X_{2}-0.6029X_{3}+0.3526X_{1}^{2}-0.0872X_{2}^{2}\;+0.0603X_{3}^{2}-0.3011X_{1}X_{2}+0.2464X_{1}X_{3}+0.8745X_{2}X_{3}\;$$

**Figure 3:**
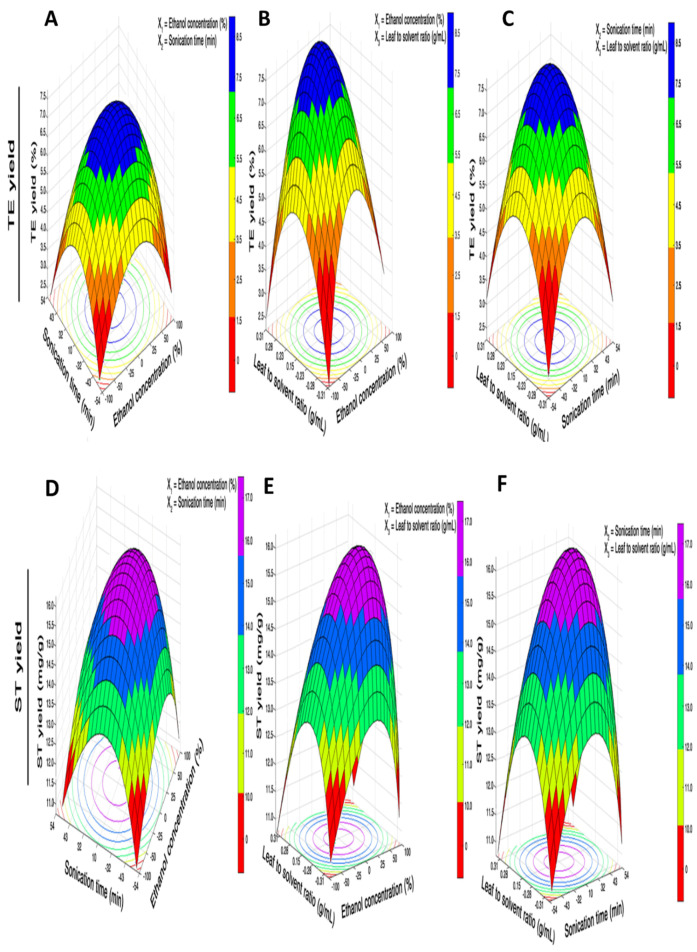

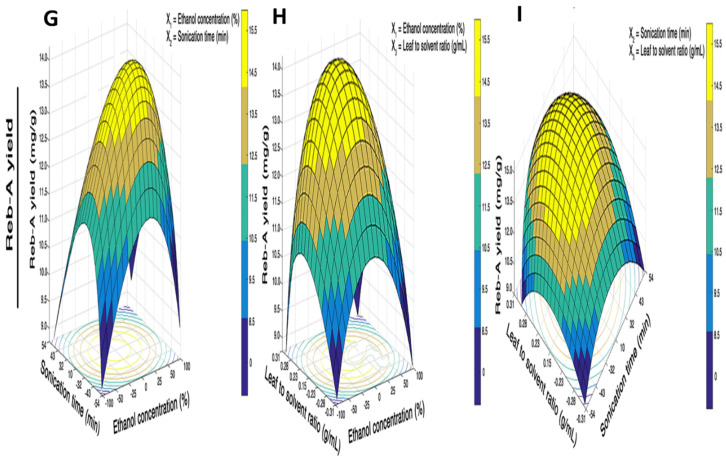
3D response surface curve and corresponding contour plots of TEY showing the interaction effect of the concentration of ethanol and sonication time at fixed level of leaf–solvent ratio (0.23 g·mL^−1^). (**A**) Concentration of ethanol and leaf–solvent ratio at a fixed level of sonication time (32 min) (**B**) and sonication time and leaf–solvent ratio at a fixed level of concentration of ethanol (50%). (**C**) ST yield showing the interaction effect of concentration of ethanol and sonication time at fixed level of leaf–solvent ratio (0.23 g·mL^−1^). (**D**) Concentration of ethanol and leaf–solvent ratio at fixed level of sonication time (32 min) (**E**) and sonication time and leaf–solvent ratio at fixed level of concentration of ethanol (50%) (**F**); Reb-A yield showing the interaction effect of concentration of ethanol and sonication time at fixed level of leaf–solvent ratio (0.23 g·mL^−1^). (**G**) Concentration of ethanol and leaf–solvent ratio at fixed level of sonication time (32 min) (**H**) and sonication time and leaf–solvent ratio at fixed level of concentration of ethanol (50%) (**I**).

The model *R*^2^ value was 0.9401 as shown in Table 3, which evidenced existing variability of the input variables which could be explicable up to 94% of the variation in the corresponding TEY. For the *Y*_1_ response, it is evident from Table 3 that a fairly high *R*^2^ value (0.9401) and non*–*significant lack of fit (0.0128) suggested adequacy of the model (Equation (10)) at the 95% confidence interval and well*–*fitting of experimental data. The highest TEY was obtained from experimental run No. 8 under the following extraction conditions: *X*_1_ of 75%, *X*_2_ of 43 min, and *X*_3_ of 0.28 g·mL^−1^. The lowest TEY was obtained at *X*_1_ of 75%, *X*_2_ of 43 min, and *X*_3_ of 0.28 g·mL*^−^*^1^. It could be observed from regression evaluation that the independent UAE process variables exhibited a linear effect on TEY. For TEY, the ethanol concentration and sonication time were found as more influential at a level of *p* < 0.01 than the leaf–solvent ratio at a level of *p* < 0.05. Conversely, quadratic terms of $X_{1}^{2}$ and $X_{2}^{2}$ were highly significant (*p* < 0.01) in comparison with that of $X_{3}^{2}$ (*p* < 0.05), while all the interaction effects were found to be statistically significant at *p* < 0.01. The experimental yield of the *Y*_1_ responses showed an increasing trend with increases in the three input variables. TEY as function of ethanol concentration and sonication time exhibited a rising tendency with a fixed level of leaf–solvent ratio at 0.23 g·mL^−1^ (Figure 3A). The response surfaces exhibited well*–*defined convexity and surface curvatures (Figure 3B,C) which suggested similar trends for TEY as functions of *X*_1_ and *X*_3_, and *X*_2_ and *X*_3_ at fixed levels of sonication time (32 min) and ethanol concentration (50%), respectively. The TEY reached a maximum value near the midpoint region of response plots. The leaf–solvent ratio worked as a vital factor in achieving an increased TEY. This could be explained in terms of localized heating of the extraction solvent owing to cavitation phenomenon during UAE, which causes mechanical disruption of the cell walls and particle collisions followed by a release of the cellular contents [30].

### 3.3. Process Variables Effect on ST Yield

The ST yield values that were obtained from the UAE extracts are given in Table 2 with their corresponding extraction conditions according to the CCD matrix. A full quadratic model equation (Equation (7)) was constructed in coded notations by using coefficients that are given in Table 3 after polynomial regression analysis.

(11) $$\begin{array}{cc} Y_{2}\;\left(mg/g\right)= & +43.1629+0.3904X_{1}+0.4993X_{2}+0.6198X_{3}+0.0313X_{1}^{2}\\ & -0.0887X_{2}^{2}+0.0756X_{3}^{2}-0.0091X_{1}X_{2}+0.0081X_{1}X_{3}\\ & +0.0448X_{2}X_{3} \end{array}$$

**Figure 4:**
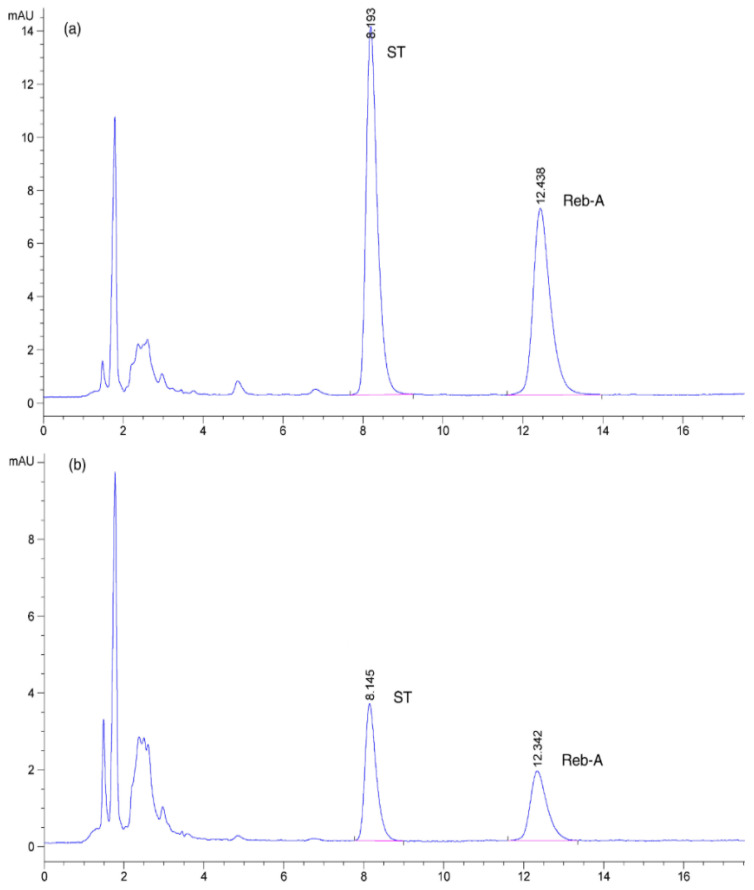
Standard HPLC chromatograms of quantified glycoside compounds including ST and Reb-A (**a**) and UAE extract (**b**) at optimized process variables of 75% ethanol concentration, 43 min sonication time, and 0.28 g·mL^−1^ leaf-to-solvent ratio.

A variation of 88.74% in the ST yield could be explained based on the model *R*^2^ value (Table 3). The experimental data were fitted well and a high *R*^2^ value and non*–*significant lack of fit (0.03561) suggested model validity (Equation (11)). The UAE extract that was obtained under the extraction conditions of run No. 8 exhibited higher ST yield (20.76 mg/g), found to be in fair match with those of the predicted yield values of 19.45 mg/g as evidenced by HPLC chromatograms which demonstrated the quantified component glycosides peaks in standard chromatographic depictions (Figure 4a) and UAE extract (Figure 4b) at optimized UAE process variables. The ST yield showed significant increases with corresponding increases in the independent variables. The role of extractant and crystallization solvents including methanol, ethanol, and isopropyl was studied by and authors who concluded that ethanol as crystallization solvent exhibited a great influence on the recovery of SGs (ST, Reb-A, and Reb-C) crystals [31]. Ethanol was reported to cause the highest recovery rate with improved purity. The 3D response surface plots (Figure 3D*–*F) demonstrated that the ST yield was affected by UAE process parameters in the same manner as TEY was affected. The sharp and higher convexity of the plots indicated optimal ranges of the independent variables resulting in maximum response values. This implied a close association between the *Y*_1_ and *Y*_2_ responses. Corresponding to our results, a correlation of the total extract recovery and glycoside yield was also reported by Jaitak, Bandna, & Kaul [8]. Regression analysis showed that *X*_2_ was significantly (*p* < 0.01) more influential among the linear terms as compared to *X*_1_ and *X*_3_. All the quadratic and interaction terms were statistically significant at a level of *p* < 0.01. Our results were endorsed by Periche et al.’s [22] findings who recovered increased ST recovery (47 mg/g) by means of UAE at a sonication time of 20 min in comparison with the conventional extraction (29 mg/g) by thermostatic bath at atmospheric pressure.

### 3.4. Process Variables Effect on Reb-A Yield

The Y_3_ mean response values are provided in Table 2, and quadratic model equation that was generated from regression analysis in coded form is given below as shown in Equation (12).

(12) $$\begin{array}{cc} Y_{3}\;\left(mg/g\right)= & +19.1186-0.1551X_{1}-0.2916X_{2}-0.3228X_{3}+0.0726X_{1}^{2}\\ & -0.0593X_{2}^{2}+0.0323X_{3}^{2}-0.0211X_{1}X_{2}+0.0664X_{1}X_{3}\\ & -0.6946X_{2}X_{3} \end{array}$$

A high *R*^2^ (0.9758) and non*–*significant lack of fit (0.00517) suggested model validity for Reb-A yield at 95% confidence interval. The highest yield of Reb-A glycoside (16.45 mg/g) was obtained from run No. 8 at specified conditions (Table 2), and an experimental yield value as well as predicted Reb-A yield value exhibited a fair match. All the linear and quadratic terms significantly (*p* < 0.01) affected the *Y*_3_ response, while the interaction of *X*_1_ and *X*_2_ affected *Y*_3_ more significantly (*p* < 0.001) as compared to other interaction terms (*p* < 0.01). Similar to the *Y*_1_ and *Y*_2_ responses, 3D response plots demonstrated that the *Y*_3_ response showed positive correlation in a linear fashion with corresponding increases in the independent variables as evidenced from the convex nature of the response plots (Figure 3G*–*I) that were generated by plotting the *Y*_3_ response values against the two independent variables while keeping the third parameter at a fixed level. A maximum Reb-A yield was obtained near the midpoint region. The solid*–*liquid ratio exhibited significant influence on recovery yield of Reb-A and the solid*–*liquid ratio may be effective up to certain extent whereas further rises may cause increased solubility leading to reduced recovery of Reb-A [32]. Similarly, Periche et al. [22] reported an increased Reb-A yield from UAE and confirmed improved efficiency of the method as compared to conventional extraction.

### 3.5. Hybrid ANN–GA Modeling

**Figure 5:**
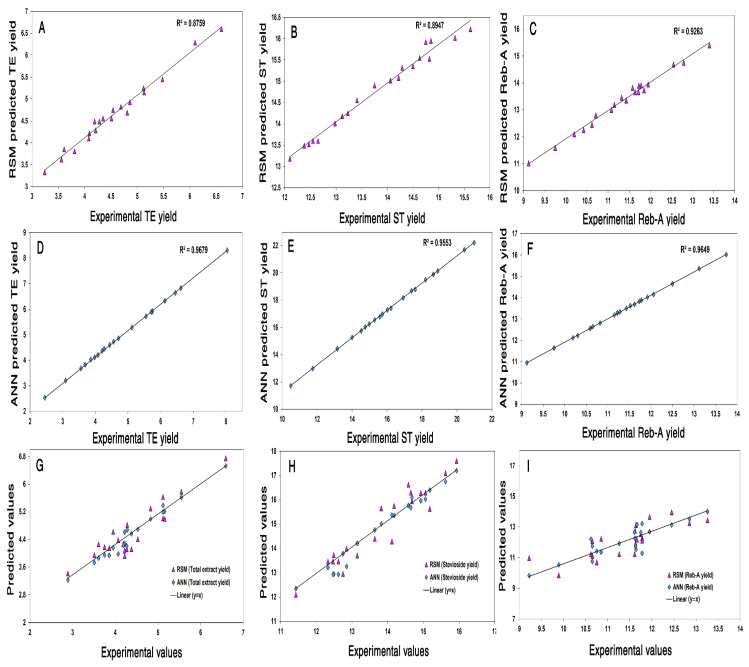
Comparison between the experimental values and model*–*predicted data values that were rendered by the RSM model for: TEY (%) (**A**) ST yield (mg/g) (**B**) and Reb-A yield (mg/g) (**C**); ANN model for: TEY (%) (**D**), ST yield (mg/g) (**E**), and Reb-A yield (mg/g) (**F**), Scatter plot showing the distribution along the straight line of predicted values versus the experimental values that were obtained by ANN and RSM models for the prediction of: TEY (%) (**G**), ST yield (mg/g) (**H**), and Reb-A yield (mg/g) (**I**).

Recently, ANN has gained popularity as a powerful simulation and optimization tool for extraction processes owing to the powerful predictive and estimation capabilities. Analogous to the human brain, ANN can be used successfully to map non*–*linear relationships between independent and dependent variables by training and constructing an ANN model [1,30]. Therefore, an ANN model was developed to trace the nonlinear relationship between the input process variables (*X*_1_, *X*_2_, *X*_3_) and the required target responses (*Y*_1_, *Y*_2_, *Y*_3_) through a topology optimization procedure involving feed*–*forward back propagation, also known as the Levenberg*–*Marquardt (LA) algorithm and it was constructed by exploiting the experimental data from CCD*–*matrix comprising of three layers: input layer, hidden layer, as well as an output layer. In this study, the number of neurons in both the input and output layers were defined by the CCD. Therefore, a selection of an appropriate number of neurons iteratively was restricted to only the hidden layer (layer 2). The whole dataset comprising of 20 data points was divided in a random manner into 3 sets: 14*–*points for training, 3*–*points for validation, and 3*–*points for testing subsets. The splitting of the data into training, validation, and testing subsets allowed for the estimation of predictive performance of the neural network regarding “unseen” data that were not employed for training [1]. As a criteria to measure the performance of the developed network, the least training and testing errors were employed to evaluate the network performance of the optimized ANN topology. A high Epoch number could result in over*–*fitting of the model during topology optimization [32]. Therefore, the Epochs number was kept to the lowest number to avoid this problem. Network training was performed by LA algorithm in order to achieve the best validation performances pertaining to the target responses: *Y*_1_, *Y*_2_, and *Y*_3_ at Epoch number 2, 1, and 4, respectively (Figure 2B–D). Various feed*–*forward neural networks (FFNNs) comprising of variegated topologies were subjected to training for establishing the neuron number in the hidden layers and the best optimized topology selection on the basis of performance criteria of the highest R^2^ and the lowest RMSE values as measures of better precision and reliability. Owing to the particular criteria, the best FFNN topologies were chosen for three target responses as given ahead; *Y*_1_: TEY (3:8:1), *Y*_2_: ST yield (3:10:1), and *Y*_3_: Reb-A yield (3:7:1), representing the neuron numbers in the three architectural layout layers comprising of input, hidden, and output, respectively. Moreover, the fair match was observed between the RSM model*−*predicted values and the observed experimental values (Figure 5A–C). Moreover, the experimental data showed good agreement with the ANN model*–*predicted data (Figure 5D–F) as evident from the high correlation values for all the response variables. All the data points were found to be in close proximity of the straight line, which indicated higher precision of the developed ANN model with respect to predictability for all the response variables for valid regions under consideration. These results suggested high predictive accuracy of the developed ANN model. For network modeling and pattern recondition, the transfer function, named hyperbolic tangent sigmoid, was employed as per the Equation (13) given below:

(13) f(x)=tansig (n)=21+e–2x −1 

The GA optimization constraints were established as given below: 

0 ≤ *X*_1_ ≤ 100

10 ≤ *X*_2_ ≤ 54

0.148 ≤ *X*_3_ ≤ 0.313

### 3.6. Predicive Performance Comparison of RSM and ANN–GA Models

For achieving better predictive modeling, the *SEP*, *RMSE*, and *AAD* values should lower. The lower *RMSE*, *AAD*, and *SEP* values in the case of the ANN*–*GA model were evident of the absolute model fit [33]. For validation and testing of the extrapolating capabilities of both models, a completely new dataset of nine runs was used (apart from the dataset that was previously employed to create the model, data not shown). Moreover, the predicted and experimental response values of both models are given in Table 2. 

The results of the statistical comparison between the RSM and ANN*–*GA models are demonstrated in Table 4. Comparative values of *R*^2^, *RMSE*, *AAD*, and *SEP* showed better performance of the ANN model with respect to generalization capability as compared to RSM. Moreover, comparative resemblance plots (Figure 5G*–*I) for the three target responses (*Y*_1_, *Y*_2_, *Y*_3_) showed that the ANN model was more precise and much better with improved accuracy for experimental data fitting in comparison with the RSM models. From the results, it was observed that the ANN model demonstrated relatively less variation with steady residuals while the RSM model exhibited larger deviations between the predicted and actual target response values, also known as residuals. The significantly higher generalization capacity of ANN could be attributed to its universal approximation ability to approximate any form of non*–*linearity/non*–*linear process behavior, whereas RSM application is effective only for quadratic non*–*linear relationship and this demands a profound insight of the defined ranges for each independent variable [34]. Similar results have been reported by Teslić et al. [35] for microwave*–*assisted extraction of polyphenols from defatted wheat germ, whereby the authors compared both RSM and ANN for their predictive modeling efficiency and compared both RSM and ANN for influence analysis, fitting quality, and optimization.

### 3.7. PCA

**Figure 6:**
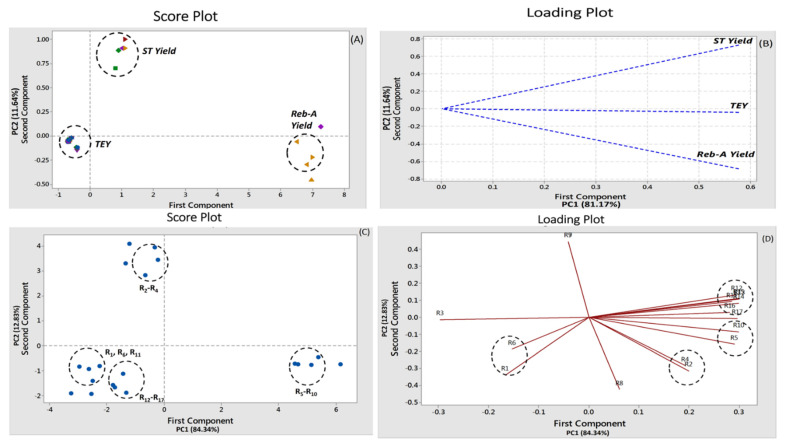
PCA score and loading plots by different target responses (**A**,**B**) and the extraction conditions (**C**,**D**). The circled clusters showed successful discrimination in terms of target responses and number of experimental runs. Different color in the PCA score plots clusters exhibited peculiarity for each target response analyzed.

PCA analysis was performed for elucidating the numerical data trend of the UAE extraction results and the effects on the target responses (TEY, ST, and Reb-A yields). PCA has been recognized as the power multivariate data analysis technique for dimensionality reduction of the multivariate data to a minimum of two to three components (PC1 and PC2) with a minimum degree of information loss. The score plots and loading plots with respect the numerical data trend for the target response and number of experimental runs are shown in Figure 6A*–*D. The original contribution to the total variance by the variables in terms of the target responses that were reached for PC1 and PC2 was up to 81.17% and 11.64%, respectively (Figure 6A,B). Eigen-analysis of the correlation matrix is provided in Table S2. The number of experimental runs also exhibited influence on the total variability, whereby PC1 and PC2 contributions to the total variance owing to number of experimental runs at CCD*–*specified conditions were 84.34% and 12.83%, respectively (Figure 6A,B). The distinct clusters were evident on the PCA score plots which indicated that the ethanol concentration and extraction time had a significant influence on the target responses (YEY, ST, and Reb-A yields). Moreover, the ST*–*yield and Reb-A yield lay on the positive side of the PCA score plot and the number of experimental runs including R*–*R4 and R5*–*R10 were positively correlated with the TEY, ST, and Reb-A yields. Therefore, it might be implied that the PCA may serve as the valuable chemometric tool to elucidate the numerical data trend for classifying information based on the stevia samples in correlation with the target responses and the UAE extraction conditions at various experimental conditions. These results were in agreement with the findings of the Choi et al. [29] who reported that PCA was successful to elucidate the numerical data trend for *Nypa fruticans* samples in correlation with the antioxidant activities.

### 3.8. Physicochemical Features and Glycosides Extraction Phenomenon

With regard to the chromatographic separation of glycosides, a hydrophilic interaction liquid chromatography (HILIC) mode was employed. In HILIC of separation, the column packing comprised of spherical silica particles (5 μm) which were covalently bonded to carbonyl groups. With the HILIC columns, the distinctive selectivity for enhanced separation of target glycosides was provided by the stationary phase (amide: NH_2_ in this case). Owing to this phenomenon, a higher degree of resolution of ST and Reb-A was achieved during chromatographic separation. Furthermore, 5 μm column was reported to exhibit improved selectivity as compared to 10 μm. In comparison with traditionally employed amino phases, the amide*–*80 column (5 μm) rendered improved selectivity and unique stability with higher peak sensitivity which enabled efficient chromatographic separation of SGs. In addition to this, a silica matrix as stationary phase precluded splitting of SGs peaks at a lower temperature range [36,37,38]. It was also reported in various published reports that the application of amino (NH_2_*−*)–bonded columns led to the efficient separation of SGs (ST and Reb-A) under HILIC separation mode and isocratic elution in comparison with conventional reverse*–*phase (RP) columns [37,38]. A poor degree of selectivity has been reported in the case of RP columns as far as glycoside separation including for ST and Reb-A, whereas the amino*–*bonded column (TSKgel NH_2_–80) exhibited a higher degree of efficiency owing to its hydrogen retention mechanism which caused bonding between the carbonyl group of the stationary phase (amide) and hydroxyl groups of the sample [36]. Moreover, a specified flow rate (1 mL·min^−1^) achieved enough contact time between the carbonyl groups and SGs molecules to acquire a maximum degree of separation along the silica matric layer with an improved level of selectivity. 

Furthermore, ethanol as a polar extracting solvent resulted in structural changes in the cellular matrix of the leaf powder which is attributed to the intra*–*crystalline and osmotic swelling. The led to enhanced solubilization along with mass transfer of the target analyte SGs components including ST and Reb-A to solution because of the disruption of the binding of the matrix and analyte. UAE is a highly effective extraction technique to recover active principles from plant sources due to the cavitation phenomenon [39]. After exposure to ultrasonic waves, cavitation bubbles are formed near the interface boundary between the extraction solvent (ethanol) and the solid plant matrix. Cavitation also results in enhanced mass transfer and extraction kinetic rates due to a localized rise in temperature at the interfacial region. This phenomenon produces two*–*fold effects: (1) localized heating of the solvent causes mechanical disruption of the cell walls followed by a release of the cellular contents, and (2) increased diffusion rate renders higher extract yields [14,39]. Moreover, the target compounds after dissolution reached the interfacial region that existed between the extracting solvent (ethanol in this case) and the sample matrix (finely ground particles in powdered form) which facilitated the mass transfer and led to maximum dissolution of SGs in bulk solution [40].

### 3.9. Comparison of Extraction Efficiencies of UAE and CME

**Figure 7:**
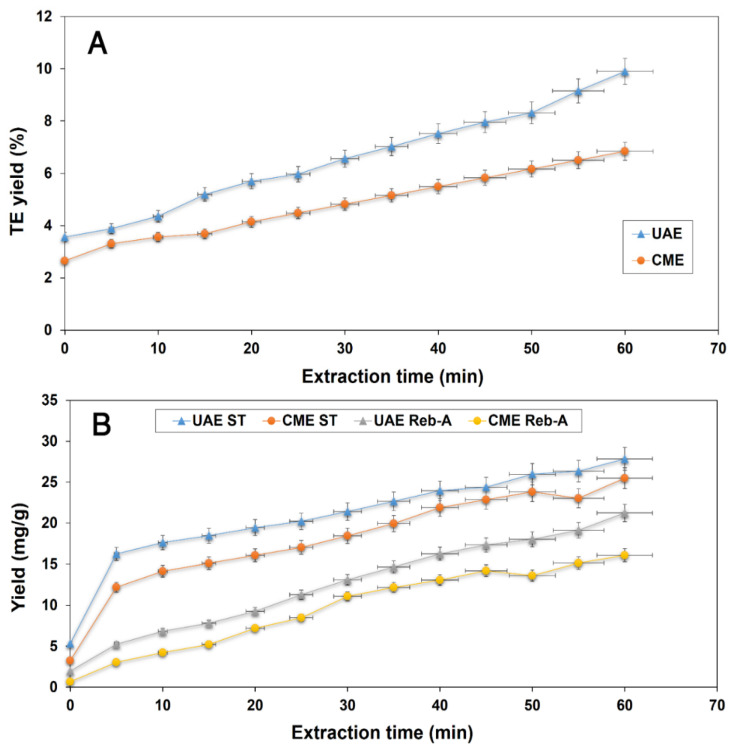
UAE comparison at optimized extraction conditions (*X*_1_: 75% ethanol concentration, *X*_2_: 43 min sonication time and *X*_3_: 0.28 g·mL^−1^ leaf-to-solvent ratio) and CME for: TEY (%) (**A**), ST, and Reb-A yields (mg/g) (**B**).

For the estimation and validation of the efficiency of ultrasound on SGs extraction from stevia leaf powder, both ecofriendly sonication (UAE) and conventionally employed (CME) methods were subjected to comparison, and the results are demonstrated in Figure 7. It was evidenced from the results that the UAE method rendered a higher recovery of the target responses including TEY, ST, and Reb-A yields at optimized extraction conditions in accordance with the CCD specification as compared to that of the recoveries from the CME (24 h) procedure as far as efficiency is concerned. A reduced extraction time, energy, and solvent consumption were some of the chiefly rendered advantages that were gained by utilizing the UAE as an alternative to CME in conjunction with a higher recovery of the desired bioactive component*–*rich extracts from stevia plant matrix. A high yield rate from the UAE procedure in shorter times can be explained by the cavitation phenomenon that results in a collapse of bubbles during the exposure of waves near the matrix interfaces, which causes a rupturing of the cell structure followed by an enhanced mass transfer of the extractable components to the extraction solvent [41]. Šic Žlabur et al. [23] have reported UAE to be more rapid and efficient for ST and Reb-A extraction; conventional hot water extraction aided with magnetic stirring required 24 h to yield 74 mg/g ST and 22 mg/g Reb-A while UAE rendered higher recoveries of both ST (96.5 mg/g) and Reb-A (37 mg/g) in 10 min using a probe diameter of 22 mm. Similarly, Alupului et al. [7] have also reported comparable yields of ST and Reb-A from both UAE and conventional solvent extraction; UAE proved to be more effective and simpler and required only 20 min as compared to the 24–h conventional extraction. Corresponding to our results, Jaitak, Bandna, and Kaul [8] have also reported UAE to be more efficient and rapid for steviol glycoside extraction compared with the 12–h conventional cold extraction.

There were two more comparative parameters that were also employed including energy consumption and CO_2_ emission to compare the UAE and CME efficiency. The energy consumption and CO_2_ emission calculations were carried out as per the specified revised guidelines of IPCC [42]. The power and time were subjected to multiplication to calculate the power consumption in terms of kWh. Furthermore, the energy consumption calculation was also performed to calculate the TOE (tonne of oil equivalent) in accordance with the Equations (14) and (15) given below, which took into account the fuel calorific value, as specified by the Republic of Korea Energy Act [43]; implying total calorific value/1 kWh electricity use that was equivalent to 2300 kcal. Power consumption was subjected to conversion to the CO_2_ emissions (Tonnes CO_2_: TCO_2_) by employing the factor pertaining to greenhouse gas emissions (0.4585 TCO_2_ equivalent/ MWh) as notified by the Korea Power Exchange [44] and given in Equation (16).

Power consumption (kWh) = Power (W) × time (h)(14)

Energy consumption (TOE) = fuel calorific value kcal/10^7^ (15)

CO_2_ emissions (TCO_2_ equivalent) = power consumption × greenhouse gas emissions factor × 1000 (16)

**Figure 8:**
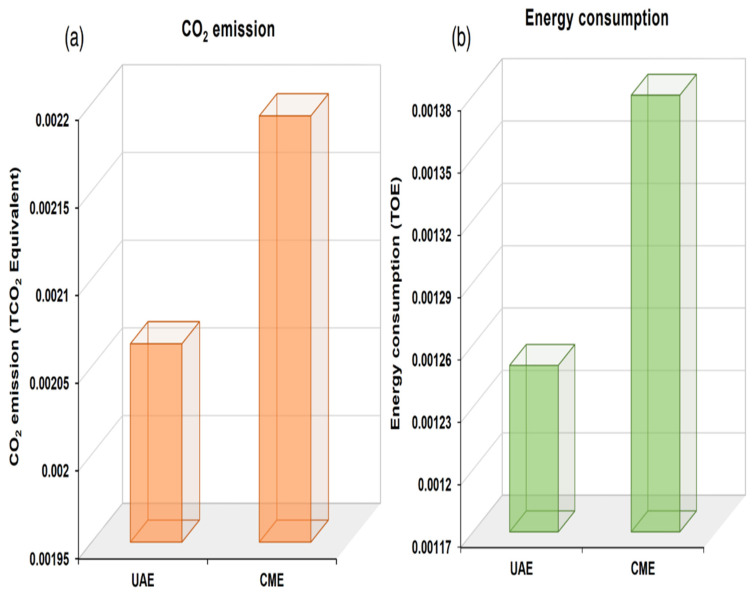
Efficiency comparison on the energy consumption (**a**) and CO_2_ emission (**b**) from the UAE and CME extraction methods.

The depiction of the results pertaining to the CO_2_ emissions and energy consumption is given in the Figure 8. Moreover, the UAE exhibited relatively lower amounts of CO_2_ emissions (0.000023 TCO_2_ equivalent) as compared to that which was calculated for CME (0.0028 TCO_2_ equivalent). Further, lower CO_2_ emissions (1/120), reduced time consumption (1/100), and energy utilization (1/110) were exhibited by the eco*–*friendly UAE method. It was endorsed by these results that the UAE method was found to be adequately suitable to extract bioactive component*–*rich stevia leaf powder extract with reduced resource consumption in comparison with the CME method.

## 4. Conclusions

In the current research, both the RSM and ANN modeling approaches were employed to determine the optimum UAE extraction conditions that yield maximum TE, SGs including ST and Reb-A yields from stevia (*S. rebaudiana*) leaf powder. A comparative overview of both modeling techniques based on assessment using *R*^2^, *RMSE*, *AAD*, and *SEP* parameters demonstrated the superiority of the ANN–GA model over RSM. Therefore, it can be concluded that even though the optimization of the extraction processes is most widely performed using RSM, the hybrid ANN–GA technique could be employed as a better alternative with improved accuracy and predictive capability. Moreover, the requirement of a lower number of experimental runs that are independent of experimental design makes hybrid ANN–GA a preferred choice for efficient and optimum UAE of SGs from stevia leaves as compared to RSM. A PCA was highly effective to elucidate the numerical data trend for the target responses and effects of the experimental runs at specified conditions. Finally, the optimum and economic UAE parameters resulting in the maximum target responses were *X*_1_ of 75%, *X*_2_ of 43 min, and *X*_3_ of 0.28 g·mL^−1^, which could be implemented to scale−up at an industrial level. Moreover, in comparison with the CME method, higher TE, ST, and Reb-A recoveries were achieved through UAE with reduced consumption of resources and CO_2_ emission. Additionally, the UAE method may serve as the eco–friendly method with improved efficiency as an alternative to the conventionally employed maceration extraction to extract the bioactive component–rich extract, exhibiting higher amounts of SGs including ST and Reb-A from stevia leaf powder.

## Figures and Tables

**Table 1:** UAE process parameters with their particular experimental ranges and levels.

Input Variable	Variable Range and Levels (Coded)						
	Unit	Code	−1.68 (−α)	−1	0	1	1.68 (+α)
Ethanol concentration	%	*X*_1_	0	25	50	75	100
Sonication time	min	*X*_2_	10	21	32	43	54
Leaf to solvent ratio	g/mL	*X*_3_	0.148	0.18	0.23	0.28	0.313

**Table 2:** CCD-specified experimental design with target responses as a function of the independent UAE process variables.

				Dependent Variables								
Run No. ^2^	Independent Variables ^1^			*Y*_1_: TE Yield (%)			*Y*_2_: ST Yield (mg/g)			*Y*_3_: Reb-A Yield (mg/g)		
	*X*_1_ (%)	*X*_2_ (min)	*X*_3_ (g/mL)	Experimental Data	RSM Predicted	ANN Predicted	Experimental Data	RSM Predicted	ANN Predicted	Experimental Data	RSM Predicted	ANN Predicted
1	25 (−1)	21 (−1)	0.18 (−1)	5.67 ± 0.07 ^3^	5.85	5.80	14.42 ± 0.04	15.66	15.04	10.96 ± 0.16	12.56	13.04
2	25 (−1)	21 (−1)	0.28 (+1)	6.01 ± 0.04	6.13	6.09	14.63 ± 0.02	15.12	15.81	12.85 ± 0.03	13.45	15.81
3	25 (−1)	43 (+1)	0.18 (−1)	6.03 ± 0.05	6.34	6.12	14.63 ± 0.02	15.90	15.97	12.85 ± 0.03	13.52	15.96
4	25 (−1)	43 (+1)	0.28 (+1)	7.37 ± 0.03	7.21	7.56	16.94 ± 0.04	17.85	17.32	13.97 ± 0.03	12.75	13.57
5	75 (+1)	21 (−1)	0.18 (−1)	6.02 ± 0.03	6.57	6.16	14.64 ± 0.04	14.12	15.45	12.85 ± 0.04	13.51	14.41
6	75 (+1)	21 (−1)	0.28 (+1)	6.74 ± 0.05	6.61	6.87	16.44 ± 0.03	16.15	17.12	13.34 ± 0.03	14.87	14.57
7	75 (+1)	43 (+1)	0.18 (−1)	7.27 ± 0.03	7.05	7.39	16.85 ± 0.02	18.32	17.86	13.87 ± 0.02	12.42	14.47
8	75 (+1)	43 (+1)	0.28 (+1)	8.85 ± 0.05	8.93	9.86	20.76 ± 0.03	19.45	21.81	16.45 ± 0.04	17.03	17.22
9	50 (0)	32 (0)	0.23 (0)	6.47 ± 0.05	6.04	6.57	16.86 ± 0.04	17.94	17.15	13.86 ± 0.02	15.12	17.15
10	50 (0)	32 (0)	0.23 (0)	6.46 ± 0.03	6.13	6.49	16.87 ± 0.03	17.89	17.33	13.87 ± 0.04	15.09	17.26
11	0 (−α)	32 (0)	0.23 (0)	5.81 ± 0.02	6.47	5.71	14.55 ± 0.03	15.64	15.12	12.46 ± 0.03	13.56	15.01
12	100 (+α)	32 (0)	0.23 (0)	8.01 ± 0.04	7.80	7.57	16.95 ± 0.04	18.13	17.75	13.94 ± 0.03	13.51	15.86
13	50 (0)	10 (−α)	0.23 (0)	5.99 ± 0.04	6.46	6.09	14.65 ± 0.04	13.90	15.14	12.85 ± 0.03	13.47	14.03
14	50 (0)	54 (+α)	0.23 (0)	7.35 ± 0.03	7.79	7.37	16.93 ± 0.04	17.47	17.86	13.97 ± 0.03	15.29	15.87
15	50 (0)	32 (0)	0.148 (−α)	5.57 ± 0.03	5.65	5.47	14.46 ± 0.03	15.66	15.12	11.94 ± 0.06	13.14	14.45
16	50 (0)	32 (0)	0.313 (+α)	6.94 ± 0.02	7.11	6.89	17.03 ± 0.04	17.80	17.59	14.02 ± 0.04	15.26	14.87
17	50 (0)	32 (0)	0.23 (0)	6.43 ± 0.03	6.01	6.56	16.86 ± 0.04	17.92	17.12	13.85 ± 0.02	15.07	17.11
18	50 (0)	32 (0)	0.23 (0)	6.46 ± 0.03	6.09	6.52	16.85 ± 0.03	17.97	17.27	13.86 ± 0.04	15.13	17.04
19	50 (0)	32 (0)	0.23 (0)	6.45 ± 0.02	6.03	6.51	16.86 ± 0.04	17.93	17.09	13.85 ± 0.02	15.11	17.09
20	50 (0)	32 (0)	0.23 (0)	6.47 ± 0.03	6.11	6.54	16.87 ± 0.03	17.91	17.35	13.87 ± 0.04	15.16	17.21

^1^ *X*_1_: ethanol concentration, *X*_2_: sonication time, *X*_3_: leaf/solvent ratio. ^2^ Experimental conditions in accordance with the CCD–specified design points. ^3^ Experimental values: mean ± S.D. (*n* = 3).

**Table 3:** ANOVA table showing the model terms (linear, quadratic, and interaction effects) of each variable and coefficients for model prediction.

		*Y*_1_: TE Yield (%)	*Y*_2_: ST Yield (mg/g)	*Y*_3_: Reb-A Yield (mg/g)
Source	DF	Estimated Coefficient	Estimated Coefficient	Estimated Coefficient
Model	9	282.3273 **	119.727 *	28.9583 **
Intercept				
(*β*_0_)	1	26.8068 **	43.1629 **	23.7821 **
Linear terms				
*X*_1_ (*β*_1_)	1	−0.335019 **	0.390353 *	−0.685319 **
*X*_2_ (*β*_2_)	1	0.471612 **	0.499269 **	−0.430433 **
X_3_ (*β*_3_)	1	−0.602871 *	0.619773 *	−0.471874 **
Quadratic terms				
*X*_1_^2^ (*β*_11_)	1	0.352586 **	0.031327 **	0.070971 **
*X*_2_^2^ (*β*_22_)	1	−0.087273 **	0.088718 **	−0.059163 **
*X*_3_^2^ (*β*_33_)	1	0.080291 *	0.075561 **	0.030346 **
Interaction terms				
*X*_1_*X*_2_ (*β*_1_*β*_2_)	1	–0.301062 **	−0.009164 **	−0.002171 ***
*X*_1_*X*_3_ (*β*_1_*β*_3_)	1	0.246349 **	0.008153 **	0.054853 **
*X*_2_*X*_3_ (*β_2_β*_3_)	1	0.874523 **	0.044789 **	−0.001965 **
Lack of fit(probability)	7	0.0128	0.03561	0.00517
F–value probability		<0.001	<0.001	<0.001
*R* ^2^		0.9401	0.8874	0.9758
Adj. *R*^2^		0.8641	0.8419	0.9063
Predicted. *R*^2^		0.8396	0.8128	0.8363

* *p* < 0.05, ** *p* < 0.01 and *** *p* < 0.001.

**Table 4:** Predictive comparison parameters showing the estimation comparison of the RSM and ANN models for the three target responses.

	*Y*_1_: TE Yield (%)		*Y*_2_: ST Yield (mg/g)		*Y*_3_: Reb-A Yield (mg/g)	
Parameters	RSM	ANN	RSM	ANN	RSM	ANN
*R*^2^ (%)	94.01	96.71	88.74	94.39	97.58	98.83
*RMSE*	3.72	2.05	4.53	1.58	6.54	1.71
*AAD* (%)	1.337	0.238	1.689	0.751	1.327	0.8137
*SEP* (%)	0.22	0.08	0.19	0.11	0.25	0.07

## Data Availability

Not applicable.

## Supplementary Materials

The following are available online at https://www.mdpi.com/article/10.3390/foods11060883/s1, Figure S1: Fitness value Vs Generation plot for genetic algorithm, Table S1: Setting parameters of genetic algorithm used in the optimization of process, Table S2: Eigen-analysis of the Correlation Matrix from Principal Component Analysis.
