# Supplementary material for: A Hybrid RSM-ANN-GA Approach on Optimization of Ultrasound-Assisted Extraction Conditions for Bioactive Component-Rich Stevia rebaudiana (Bertoni) Leaves Extract
Source: Foods. 2022 Mar 20;11(6):883. doi: 10.3390/foods11060883 (PMC8950805; doi:10.3390/foods11060883)
Supplement: Supplementary file 1 [file foods-11-00883-s001.zip › foods-1620766-supplementary.pdf]

Supplementary Material

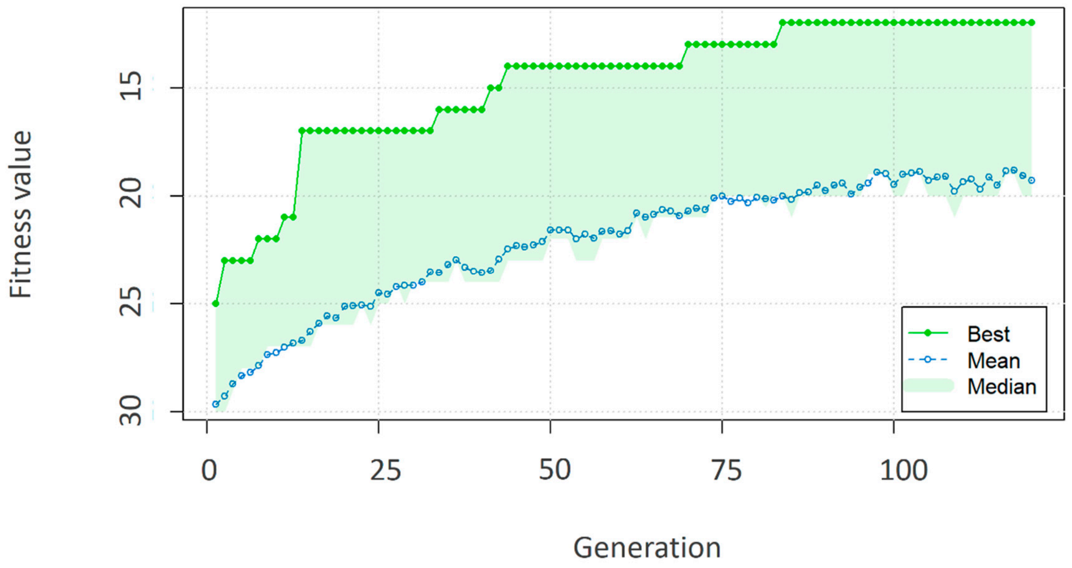

Figure S1. Fitness Value Vs generation plot for genetic algorithm.

Table S1. Setting parameters of genetic algorithm used in the optimization of process

Setting parameters	Values
Population size	50
Crossover function	Constraint dependent
Mutation function	Constraint dependent
Plot function	Best fitness
Migration	Direction (forward), fraction (0.2), Interval (20)
Scaling function	Rank
Selection function	Stochastic uniform
Elite count	0.05* population size
Nonlinear constraint algorithm	Augmented Lagrangian

Table S2. Eigenanalysis of the Correlation Matrix from Principal Component Analysis

Principal Component Analysis:

Eigenanalysis of the Correlation Matrix

Eigenvalue	10.201	4.582	1.323	0.327	0.266	0.183	0.058	0.025	0.018	0.009	0.004
Proportion	0.600	0.270	0.078	0.019	0.016	0.011	0.003	0.001	0.001	0.001	0.000
Cumulative	0.600	0.870	0.947	0.967	0.982	0.993	0.996	0.998	0.999	0.999	1.000

Variable	PC1	PC2	PC3	PC4	PC5	PC6	PC7	PC8	PC9	PC10
R1	-0.167	-0.342	0.308	-0.045	-0.290	-0.381	0.198	-0.113	-0.228	-0.536
R2	0.199	-0.315	-0.228	-0.251	-0.370	-0.183	0.363	-0.064	-0.115	0.216
R3	-0.297	-0.015	-0.154	0.045	-0.469	-0.035	-0.258	-0.288	0.386	-0.083
R4	0.192	-0.285	-0.267	0.473	0.168	-0.627	-0.196	0.228	0.198	0.085
R5	0.290	-0.159	-0.109	0.018	0.014	0.004	0.169	-0.422	-0.045	0.465
R6	-0.153	-0.188	0.654	0.241	-0.135	0.074	0.294	0.189	0.276	0.426
R7	-0.041	0.445	-0.022	0.338	-0.335	-0.104	0.004	-0.382	0.193	0.063
R8	0.061	-0.424	-0.058	0.463	-0.181	0.522	-0.248	0.124	-0.091	-0.134
R9	-0.041	0.446	-0.024	0.354	-0.260	-0.146	0.150	0.315	-0.551	0.109
R10	0.298	-0.087	-0.089	0.290	-0.065	0.258	0.186	-0.269	-0.226	-0.183
R11	0.285	0.092	-0.232	-0.186	-0.376	0.141	0.189	0.480	0.336	-0.133
R12	0.294	0.132	0.130	0.092	0.128	-0.088	0.190	-0.061	0.195	-0.305
R13	0.300	0.108	0.132	0.047	0.045	0.023	0.101	-0.051	0.160	-0.136
R14	0.299	0.083	0.174	-0.092	-0.216	-0.019	-0.189	0.194	0.104	0.012
R15	0.299	0.111	0.130	0.084	0.118	0.039	0.135	-0.130	0.103	-0.174
R16	0.281	0.028	0.367	-0.028	0.007	-0.132	-0.333	-0.095	-0.036	-0.021
R17	0.297	-0.006	0.191	-0.227	-0.275	-0.053	-0.494	-0.032	-0.262	0.166
